# Glycolytic expression in lower-grade glioma reveals an epigenetic association between IDH mutation status and PDL1/2 expression

**DOI:** 10.1093/noajnl/vdaa162

**Published:** 2020-11-27

**Authors:** Kevin B Givechian, Chad Garner, Steve Benz, Shahrooz Rabizadeh, Patrick Soon-Shiong

**Affiliations:** 1 NantOmics LLC, Culver City, California, USA; 2 Medical Scientist Training Program, Yale School of Medicine, New Haven, Connecticut, USA; 3 ImmunityBio, Inc., El Segundo, California, USA

**Keywords:** glioma, IDH mutation, immunometabolism, immune checkpoint, PDL1/2

## Abstract

**Background:**

The interplay between glycolysis and immunosuppression in cancer has recently emerged as an intriguing area of research. The aim of this study was to elucidate a potential epigenetic link between glycolysis, isocitrate hydrogenase (IDH) status, and immune checkpoint expression in human lower-grade glioma (LGG).

**Methods:**

Genomic analysis was conducted on 507 LGG samples from The Cancer Genome Atlas (TCGA). Data types analyzed included RNA-seq (IlluminaHiSeq) and DNA methylation (Methylation450K). Unsupervised clustering grouped samples according to glycolytic expression level and IDH status. Global promoter methylation patterns were examined, as well as methylation levels of LDHA/LDHB and immune checkpoint genes. Methylation data from a knock-in IDH1^R132H/WT^ allele in HCT116 cells and ChIP-seq data from immortalized human astrocytes using an inducible IDH1^R132H^ mutation were also assessed.

**Results:**

Glycolytic expression distinguished a tumor cluster enriched for wild-type IDH and poorer overall survival (*P* < .0001). This cluster showed lower levels of LDHA promoter methylation and a higher LDHA/LDHB expression ratio. These samples also displayed lower PDL1/2 promoter methylation and higher PDL1/2 expression, which was more pronounced for PDL2. IDH1^R132H/WT^ cell line data showed that induced changes in methylation were enriched for genes involved in immune regulation, and ChIP-seq data showed that promoter H3K4me3 decreased for LDHA, PDL2, and PDL1 upon induction of IDH1^R132H^.

**Conclusions:**

These results suggest a previously unrecognized epigenetic link between glycolysis and immune checkpoint expression in LGG. This work advances our understanding of glioma genomics and provides support for further exploration of the metabolic-immune interface in LGG.

**Key Points**

- Glycolytic expression differentiates wild-type IDH status in LGG and associates with immune checkpoint expression.
- Elevated promoter methylation in LDHA and PDL1/2 (hallmark genes of Warburg effect and immune checkpoint expression, respectively) is specific to a glycolytic-low LGG subtype enriched for IDH mutants.

**Importance of the Study**

LGG is a highly heterogenous disease in which wild-type IDH status is associated with a very unfavorable patient prognosis. This highlights the need to explore new therapeutic strategies, particularly for patients with wild-type IDH. However, the relationship between IDH status and other mechanisms of tumor progression remains poorly understood. Glycolytic expression and immune checkpoint expression have individually emerged as promising targets in a variety of tumor types but have not yet been assessed concomitantly in association with IDH mutation status within LGG. Using human glioma genomic data, we demonstrate that a strong association exists between wildtype IDH, glycolytic/Warburg expression, and PDL1/2 expression, which may help explain the unfavorable prognosis of wild-type IDH patients. Moreover, considering the epigenetic 2HG activity of IDH mutants, we extend this association to propose a methylation-regulated mechanism of glycolytic/Warburg expression and PDL1/2 expression in LGG. These findings are first to show such a relationship and may suggest the preclinical therapeutic rationale for the dual inhibition of glycolytic/Warburg and PDL1/2 expression in wild-type IDH LGG.

Brain lower-grade and intermediate-grade glioma (LGG) is defined as a grade I, II, or III malignancy with a highly variable but often poor prognosis.^1,2^ While phenotypically less aggressive than the further progressed glioblastoma (GBM), lower-grade gliomas represent a disease in certain need of further exploration to advance treatment options and prolong patient survival.^2,3^ Within LGG, tumors are often pathologically diagnosed according to genetic status of isocitrate hydrogenase 1 and 2 (IDH), where IDH-wild-type (IDH-wt) is associated with significantly worse patient prognosis.^2–4^ This biomarker remains a highly useful prognostic predictor of LGG patient survival, but further work is needed to elucidate the molecular pathways that associate with IDH-wt patients in hopes of advancing our understanding of LGG biology.^5,6^

Mechanistically, IDH mutations have been shown to cause a direct enzymatic structural change that drives production of the 2-hydroxyglutarate (2HG), an oncometabolite believed to represses the activity of DNA demethylases via TET2 inhibition.^7–10^ This, in turn, has been shown to have key epigenetic influences on the glioma methylome, leading to a global increase in DNA methylation and corresponding presentation of a glioma-CpG island methylator phenotype (G-CIMP).^11,12^ Moreover, IDH mutations likely occur early in gliomagenesis,^1,13^ rendering it possible that the resulting widespread epigenetic alterations may themselves influence gene expression and affect pathway activity.^14^

Glycolysis is a crucial metabolic pathway that drives the breakdown of glucose to pyruvate, thereby providing cells with ATP to supply cellular processes.^15^ Aggressive cancer cells often exploit the glycolytic pathway in the presence of oxygen to undergo a process of aerobic glycolysis, characterized as the Warburg Effect.^15,16^ This phenomenon is associated with increased invasiveness and drug resistance across various tumor types.^17^ Within GBM, several studies have suggested the potential of considering glycolytic and Warburg related properties to explore potentially novel therapeutic strategies, such as suppressive targeting of lactate dehydrogenase A (LDHA) thereby inhibiting the conversion of pyruvate to lactate.^18–20^ Studies examining LDH isoforms have also shown that induction of LDHA and a decrease in lactate dehydrogenase B (LDHB) are required for full activation of glycolysis and secretion of lactate.^21^ However, despite IDH being a critical enzyme in the process of metabolic regulation within the glucose-rich glial environment,^22,23^ studies in LGG have not yet interrogated the potential link between glycolysis and other potentially targetable mechanisms such as immunosuppression.

The expression of immune checkpoint expression genes has been associated with a more aggressive phenotype leading to worse patient prognosis.^24–27^ To this end, pharmacological targeting of immunosuppressive signaling (eg, via PDL1/2, PD1) has emerged over recent years as a therapeutic avenue of high clinical promise,^27^ but its overt potential in glioma has not yet been shown.^28^ Moreover, in several cancer types, the field of immune checkpoint signaling has surfaced an encouraging link between metabolic regulation and immunosuppression to clarify the complex crosstalk within the tumor microenvironment.^29–32^ In addition, studies have indicated that epigenetic mechanisms behind both glycolysis^33^ and immunosuppression may be at play.^34,35^ Because LGG has the highest frequency of IDH mutation which is known to drive widespread epigenetic changes,^36^ we were further encouraged to examine the genomic and epigenomic association between glycolysis, IDH mutation status, and immune checkpoint expression in LGG.

In this study, we sought to explore whether there exists a link between glycolysis and immune checkpoint expression in LGG, and whether this may be associated with the epigenetic consequences of IDH mutational status. We find that glycolytic expression is a strong predictor of IDH mutation status and reveals a distinct co-methylation landscape. Interestingly, we also show that PDL1/2 promoter hypomethylation and expression are strongly associated with a glycolytic-high phenotype, layering a potential epigenetic immunosuppressive explanation for the poorer prognosis of IDH-wt glioma patients. These findings are first to surface an epigenetic association for PDL1/2 expression that tracks with the IDH-wt status and elevated glycolytic expression in human glioma samples. This work may also encourage future concurrent exploration of glycolytic and immunosuppressive signaling via PDL1/2 in the context of LGG.

## Methods

### Patient Genomic Data Selection

The patient genomic data analyzed included all patients of the brain lower-grade glioma (LGG) cohort from TCGA with available mutation and gene expression data. This provided a total of 507 LGG patient tumor samples, which was initially randomly split into a discovery (*n* = 254) and validation (*n* = 253) data set. Each set was equally proportioned with identical mutation frequency of IDH1 and IDH2 (IDH) to reflect the appropriate mutation frequency of IDH in the overall LGG population. To do this, the frequency of IDH1/2 mutations was first examined in the entire TCGA LGG dataset. This revealed that ~82% of LGG patients harbored an IDH1/2 mutation. Therefore, we ensured that 82% of both the discovery set and validation set were samples with IDH1/2 mutations so that IDH1/2 mutations were not enriched in one dataset compared to the other. IDH mutation status was assessed in the LGG cohort using cbioportal (https://www.cbioportal.org).^37^ RNA-seq data were downloaded from xenabrowser (https://xenabrowser.net) and used to examine gene expression across all 507 patient samples of interest (dataset: gene expression RNAseq – IlluminaHiSeq, dataset ID: TCGA.LGG.sampleMap/HiSeqV2, unit: log2(norm_count+1)). Methylation data was also downloaded from xenabrowser (dataset: DNA methylation – Methylation450k, dataset ID: TCGA.LGG.sampleMap/HumanMethylation450, unit: beta value, platform: Illumina Infinium HumanMethylation450). Promoter methylation sites assessed for each gene were selected using the value of the cg identifier outside and closest to the 5′ end of the gene using the xenabrowser heatmap visualization tool. Spearman correlation values were computed to assess the degree of correlation between promoter methylation and gene expression.

### Survival Analysis

Data used for overall survival (OS) analysis between clusters produced from k-means was downloaded from OncoLnc (oncolnc.org).^38^ These data were parsed in Python and Kaplan–Meir plots were then produced in R. The coxph survival function in R (survival/survminer package in R) was used for significance analysis, hazard ratios, and 95% confidence intervals (*P* < .05 was considered significant).

### Gene Expression Clustering and Pathway Enrichment Analysis

K-means clustering is a popular vector quantization method that we used to cluster all patient tumor samples (*k* = 2) in the study via the ComplexHeatmap package in R using a 23-gene set. This gene set was produced by first combining the KEGG_GLYCOLYSIS_GLUCONEOGENESIS, KEGG-CITRATE_CYCLE_TCA_CYCLE, and the KEGG_OXIDATIVE_PHOSPHORYLATION gene sets together into gene set containing 230 total genes, and then taking the genes in the top 10% of highest variation across the discovery data set (highest standard deviation). This yielded the 23-gene set used as our feature set for *k*-means clustering. We used only the genes with highest expression variability because we hypothesized that the clinical and molecular variability of LGG would be reflected strongly in the high fold-change expression variability of the genes themselves; that is, the metabolic genes with high expression variability would be those that best capture the phenotypically alternative contexts, as has been shown in other tumor types.^39^

**Figure 1.**
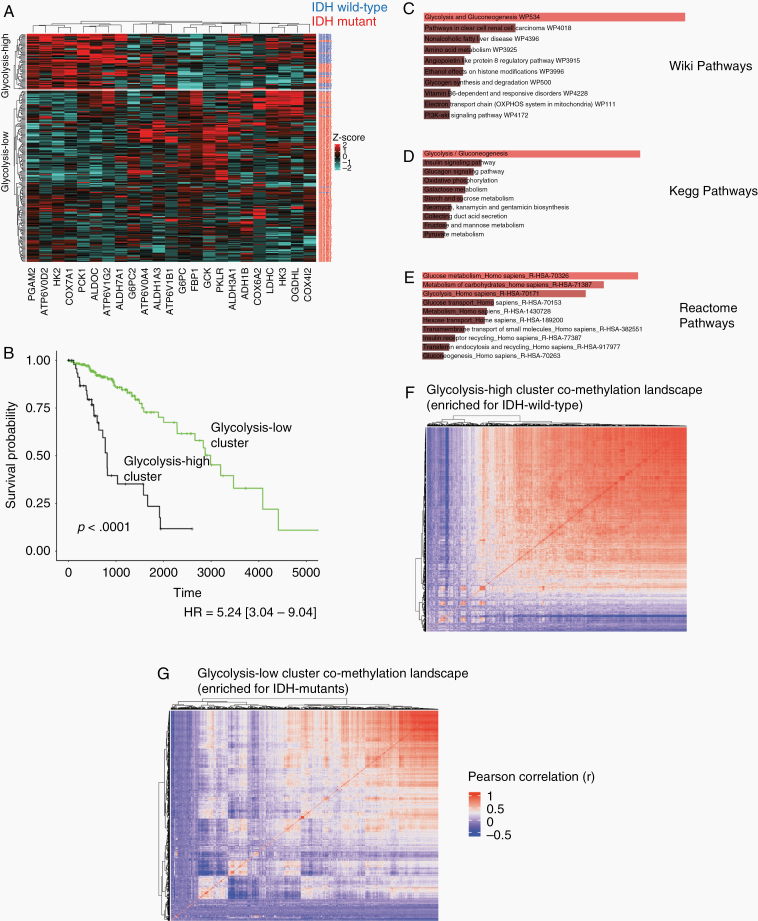
Glycolytic expression predicts patient isocitrate hydrogenase (IDH) status and alternative co-methylation landscapes. (A) Heatmap of patient clusters produced using 23 metabolic genes (blue row labels = IDH wild-type samples; red labels = IDH mutant samples). The glycolysis-high cluster is significantly enriched for IDH-wild type patients (*P* < .0001, *x*^*2*^ = 154.89; Figure 2A). (B) Overall survival (OS) analysis between clusters shows the glycolysis-high cluster patients are associated with significantly worse OS (*P* < .0001, HR = 5.24 [3.04–9.04]). (C–E) The 23 gene set was significantly enriched for the glycolysis pathway across 3 pathway databases (C (top): Wiki pathways: Adj. *P* = 3.30e−20; D (middle): Kegg pathways: Adj. *P* = 2.93e−25; E (bottom): Reactome pathways: Adj. *P* = 1.48 e−11; Figure 2C–E). (F–G) Co-methylation heatmap clustering (Pearson correlation values) of CpG cites for the glycolysis-high cluster (F) and the glycolysis-low cluster (G). The glycolysis-high cluster (IDH wild-type enriched) appeared more uniformly correlated while cluster2 (IDH mutant enriched) presented more heterogeneity across methylation sites. Red-associated dendrograms indicate sets of methylation probes that are tightly correlated together across samples, while blue-associated dendrograms indicate sets of methylation probes with inverse correlations.

This 23-gene set served as the feature set we used for k-means clustering (Figure 1) (for clustering visualization, the expression of each gene was scaled to the *z*-score relative to expression of that gene across all tumor samples analyzed in each heatmap). As assumed by the unsupervised classification process, IDH mutation status labels were not included as input features. The “N-1” Chi-squared test was used to determine statistical proportional significance of IDH mutation status enrichment between clusters.^40,41^ Together, the input dataset consisted of a table with TCGA patient IDs as rows and 23 genes as columns (23 highly variable metabolic genes) of *z*-score scaled expression values. These procedures were replicated for the patients of the validation data set using the same 23-gene set surfaced from the discovery set. We evaluated several other values of *k* (*k* = 3, *k* = 4, and *k* = 5) using the same gene set and obtained similar results. One cluster was consistently glycolytic-high (IDH wild-type enriched) with ascending *k*, while the other clusters were consistently IDH mutant enriched (data not shown). Cluster assignment labels for each sample are available upon request, and correlated grade, histology, and 1p/19q codeletion data for each patient are available at cbioportal.org.

To determine whether one pathway was more represented by these 23 genes than another pathway (eg, if glycolytic genes were more represented than TCA cycle or oxidative phosphorylation genes in the new 23 gene set), we performed a pathway analysis to show that glycolysis was indeed the pathway that was most represented in the new gene set after filtering from 230 to 23 genes. To do this, the 23 genes were run through the Enrichr pathway analysis tool to identify the enriched Kegg pathway associated with the 23-gene set (adjusted *P* < .05 was considered significant). Pathway bar plots proportional to significance enrichment level were obtained using the Enrichr tool.^42^

### DNA Methylation Analysis

Available Methylation 450k data was downloaded as described above and was used to produce a correlation matrix for visualization. Methylation 450k data was used to produce correlation matrices to compare the clustered co-methylation matrix between the glycolysis-high cluster and the glycolysis-low cluster, with clustering performed within each RNAseq cluster. The purpose of this was to determine whether global co-methylation differences between the glycolysis-high and glycolysis-low could be visualized since the glycolysis-low cluster was found to be enriched for IDH mutants. First, 485,578 CpG identifiers were narrowed down to the top 1,000 methylation sites with the highest variability (standard deviation) across the entire discovery set. Using this subset of CpG methylation values, a correlation score was computed for every possible pair of CpG sites within the samples of the glycolysis-high and glycolysis-low cluster, independently. Once these correlation matrices were obtained, heatmaps were produced of each correlation matrix for each cluster from Figure 1A (Dendograms/clustering of these matrices were done as referenced in the ComplexHeatmap package in R: https://bioconductor.org/packages/release/bioc/html/ComplexHeatmap.html). Rows and columns of the heatmaps in Figure 1F and G are CpG probes, where large red-associated dendrograms are sets of probes that are tightly correlated together across samples. The same sites with available data were then used with identical methods to visualize methylation co-enrichment arrays in the validation set.

### Immunosuppressive Gene Expression Analysis

The 9 immune genes analyzed to compare potential differences in immunosuppressive signaling genes were PDCD1LG2 (PDL2), PDCD1 (PD1), CD274 (PDL1), IDO1, TNFRSF4 (OX40), LAG3, FOXP3, TIGIT, and CTLA4.^43–49^ Patient RNA-seq expression values for each of these genes were accessed using the fbget API (https://confluence.broadinstitute.org/display/GDAC/fbget) and parsed using custom Python scripts. Two-tailed *t*-tested were used to assess which of these immune genes were differentially expressed between tumor samples of the different clusters (acceptable *P* < .0056). The Seaborn and Matplotlib libraries were used to produce and visualize scatterplots and expression swarm-boxplots for each individual gene.

## Results

### Glycolytic Expression Predicts Patient IDH Status and Alternative Co-methylation Landscapes

In order to explore whether glycolysis was enriched for a certain IDH mutational status in an unbiased manner, we first combined gene sets of the 3 main metabolic pathways (glycolysis, TCA cycle, and oxidative phosphorylation) and produced *k*-means clusters using gene expression data. Using the top 10% of most variably expressed metabolic genes from this combined gene set, 2 patient clusters were produced (Figure 1A). Cluster1 had uniformly higher metabolic expression and was significantly enriched for IDH-wild type patients (64% in cluster1 vs 3% in cluster2), whereas cluster 2 showed the opposite trend being significantly enriched for IDH mutants (*P* < .0001, *x*^*2*^ = 154.89; Figure 1A). Interestingly, the small number of IDH mutants classified to cluster1 were enriched for the 1p/19q non-codeletion molecular subtype (91% in cluster1 vs 57% in cluster2, *P* = .027, *x*^*2*^ = 4.89; Supplementary Table 1). The 23-gene signature was also prognostically significant in line with the known prognostic association of wild-type IDH, with cluster1 patients being associated with significantly worse OS (*P* < .0001, HR = 5.24 [3.04–9.04]; Figure 1B). Although surfaced from the combined glycolysis/TCA/Oxphos pathways, this gene set was significantly enriched for the glycolysis pathway across 3 pathway databases (Wiki Pathways: Adj. *P* = 3.30e−20; Kegg Pathways: Adj. *P* = 2.93e−25; Reactome Pathways: Adj. *P* = 1.48 e−11; Figure 1C–E). This therefore suggested a strong association between wild-type IDH and glycolytic expression. The overview of the study design is shown in Supplementary Figure 1.

Upon observing that cluster1 (here after referred to as the glycolysis-high cluster) and cluster2 (hereafter referred to as the glycolysis-low cluster) were IDH-wildtype and IDH-mutant enriched, respectively, we reasoned that the global methylation landscapes between clusters would be unique to each cluster. This is because mutations in IDH have been shown to drive production of 2HG and consequential epigenetic changes by inhibiting demethylase activity.^7–9^ Available data from highly variable methylation sites were used to examine these epigenetic differences, and a correlation matrix was produced for visualization. Upon clustering co-methylation scores, there appeared to be a distinct difference in the global co-methylation landscape between clusters (Figure 1F). The glycolysis-high cluster appeared to be more uniformly correlated while glycolysis-low cluster presented more heterogeneity across methylation sites (Figure 1G). This suggested that the glycolysis-high cluster and glycolysis-low cluster possessed different methylomes, in line with the demonstrated methylation altering effect of 2HG production in IDH-mutant tumors.^10,11^

**Figure 2.**
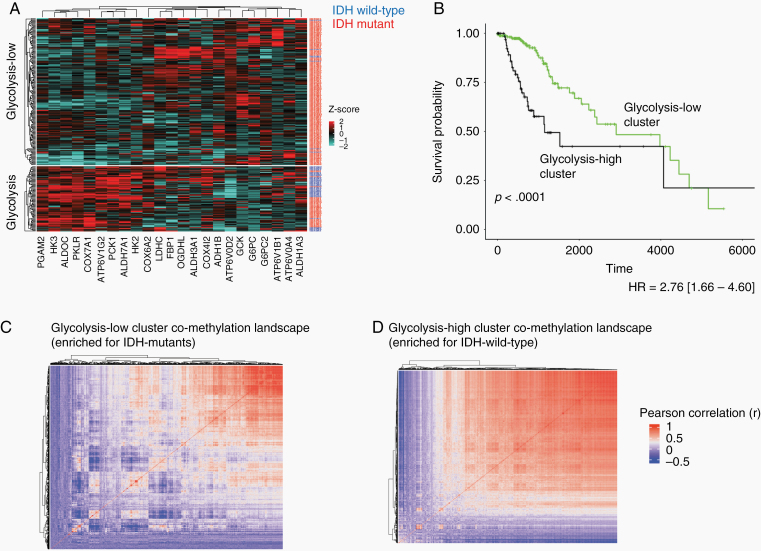
Validation set analysis showing glycolytic expression (same gene set from Figure 2) predicts patient isocitrate hydrogenase (IDH) status and alternative co-methylation landscapes. (A) Heatmap of patient clusters produced using 23 metabolic genes (blue row labels = IDH wild-type samples; red labels = IDH mutant samples). Cluster2 is significantly enriched for IDH-wild type patients (*P* < .0001, *x*^*2*^ = 76.76). (B) OS analysis between clusters shows cluster2 patients are associated with significantly worse OS (*P* < .0001, HR = 2.76 [1.66–4.60]). (C,D) Co-methylation heatmap clustering (Pearson correlation values) of CpG cites for cluster1 (C) and cluster2 (D). Cluster1 (IDH mutant enriched) displays more heterogeneity across methylation sites while cluster2 (IDH mutant enriched) appeared more uniformly correlated.

In order to confirm that the observed results were not specific to our initial dataset, we next sought to reproduce these results in a validation set. Indeed, when the same gene set was used to produce *k*-means clusters in the validation set, one cluster showed uniformly elevated expression of the glycolytic gene set and was significantly enriched for IDH-wt patient tumor samples (50% of glycolytic-high cluster samples vs 4% of glycolytic-low cluster samples; *P* < .0001, *x*^*2*^ = 76.76; Figure 2A). This result was also consistent with the reflected difference in patient OS (*P* < .0001, HR = 2.76 [1.66–4.60]; Figure 1B). The IDH mutant samples classified to the glycolytic-high cluster of the validation set were also enriched for the 1p/19q non-codeletion molecular subtype (90% in the glycolytic-high cluster vs 54% in the glycolytic-low cluster, *P* < .0001, *x*^*2*^ = 17.02; Supplementary Table 1).

Patient tumor co-methylation site clustering was also examined in the validation set. Upon visualizing the alternative methylation landscapes between the glycolysis-low cluster (IDH-mutant enriched) and the glycolysis-high cluster (IDH-wt enriched), there was a distinct difference in the global co-methylation landscape of glioma (Figure 1F), suggesting differential accumulation of 2HG.^10,11^ This observed association with glycolysis was in line with previous work,^11,12,50^ and encouraged us to examine whether this may reflect an individual epigenetic association in LDHA, a gene isoform that is critical for the Warburg Effect and the sustained activation of glycolysis.^20,21^

### Glycolytic Expression Is Associated With Elevated an Elevated LDHA/LDHB Ratio and Decreased LDHA Promoter Methylation

**Figure 3.**
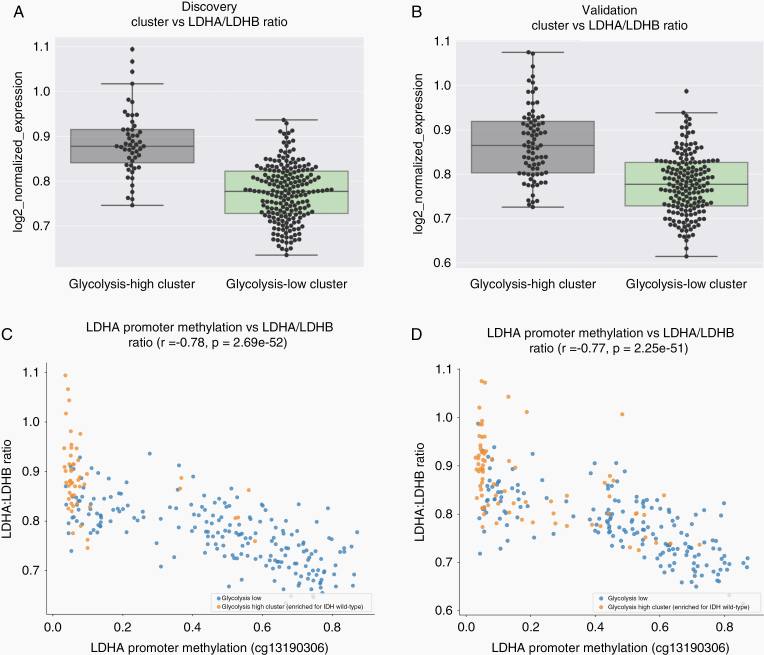
Glycolytic expression is associated with an elevated LDHA/LDHB ratio and decreased LDHA promoter methylation. (A) Boxplots showing a significantly elevated LDHA/LDHB ratio in the glycolytic-high cluster (cluster1) for the discovery set (*P* = 4.62e−15). (B) Boxplots showing a significantly elevated LDHA/LDHB ratio in the glycolytic-high cluster (cluster2) for the validation set (*P* < 0.0001). (C,D) The level of LDHA promoter methylation was also inversely associated with the LDHA/LDHB ratio in both the discovery set (C) (*r* = −0.78, *P* = 2.69e−52) and the validation set (D) (*r* = −0.77, *P* = 2.25e−51).

To examine whether the glycolytic cluster produced was associated with elevated dehydrogenase (LDH) expression, we analyzed the expression of the 2 key isoforms of LDH. The LDHA isoform converts pyruvate into lactate thereby helping sustain the Warburg effect, while the LDHB isoform catalyzes the opposite biochemical process.^20,21^ To examine whether Warburg expression was elevated in the glycolytic-high cluster, we compared the ratio of LDHA to LDHB expression between patient tumor sample clusters. We observed a significantly elevated LDHA/LDHB ratio in the glycolytic-high cluster for both the discovery and validation sets (*P* = 4.62e−15 and *P* < .0001, respectively; Figure 3A and B).

Next, we examined whether these elevated ratios of LDHA/LDHB expression may be inversely associated with LDHA promoter methylation, as increased methylation may epigenetically hinder docking of transcriptional machinery to the DNA and prevent gene expression. We found that the IDH-mutant enriched cluster showed increased levels of LDHA promoter methylation and a correspondingly lower LDHA/LDHB ratio (*r* = − 0.78, *P* = 2.69e−52; Figure 3C). This correlation was also observed in the validation dataset (*r* = − 0.77, *P* = 2.25e−51; Figure 3D). As expected, the ratio difference was also reflected by the overall levels of LDHA and LDHB expression individually (*P* = 1.54e−15, *P* = 6.09e−08; Supplementary Figure 2A and B, respectively), which was also consistent in the validation set (*P* = 1.27e−12, *P* = 6.35e−09; Supplementary Figure 2C and D, respectively). Overall, the glycolytic-high cluster, which was enriched for IDH-wild-type samples, exhibited reduced LDHA promoter methylation and a corresponding increase in the LDHA/LDHB expression ratio. This is also in line with the observed alternative global co-methylation patterns between clusters, and it reflects a biologically guided hypothesis pertaining to LDHA expression.

### Immune Checkpoint Expression Analysis Surfaces Elevated Expression and Promoter Methylation Associations for PD-L1 and PD-L2

**Figure 4.**
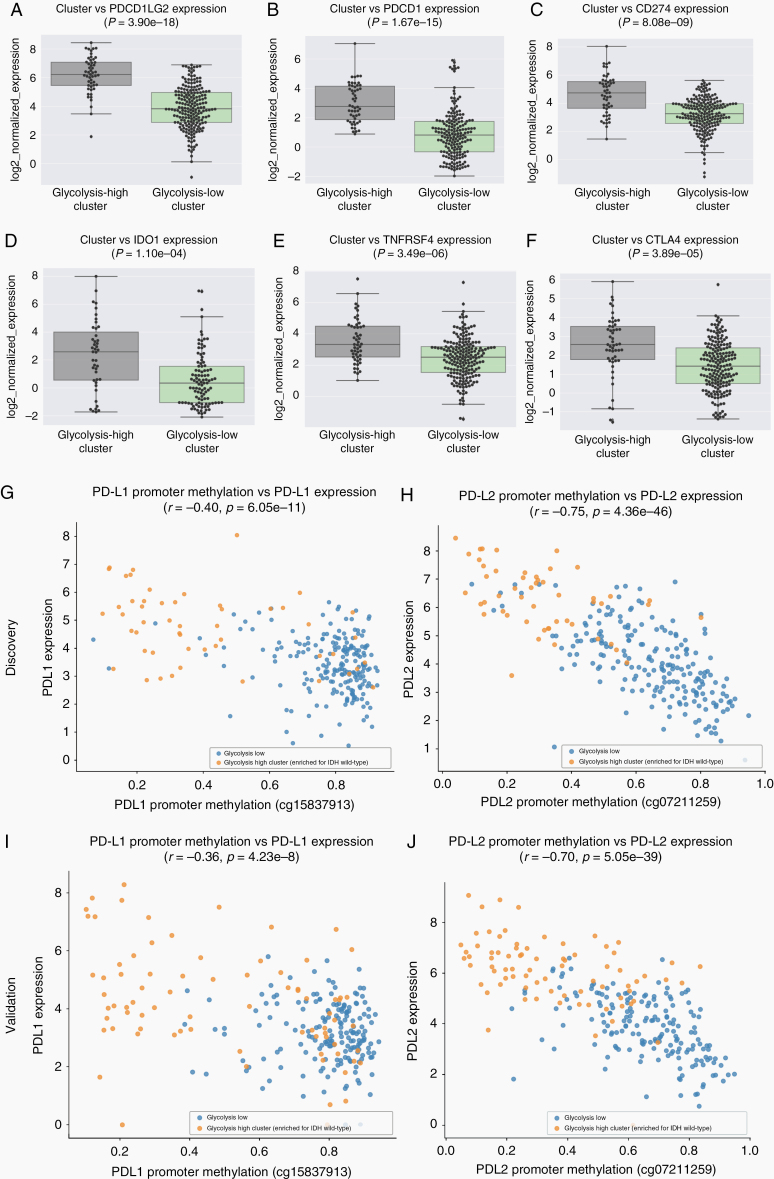
Immune checkpoint expression analysis between clusters and promoter methylation analysis. (A–F) Immune checkpoint genes with elevated expression in cluster1 (glycolysis-high, IDH wild-type enriched) were (A) PDCD1LG2 (PDL2), (B) PDCD1 (PD1), (C) CD274 (PDL1), (D) IDO1, (E) TNFRSF4 (OX40), and (F) CTLA4 (*P* = 3.90e–18, *P* = 1.67e−15, *P* = 8.08e−09, *P* = 1.10e−4, *P* = 3.49e−6, and *P* = 3.89e−05, respectively). (G) PD-L1 and (H) PD-L2 show inverse correlations between promoter methylation and gene expression (*r* = −0.40, *P* = 6.05e−11 and *r* = −0.75, *P* = 4.36e−46, respectively). (I,J) Similar results were reproduced in the validation set for (I) PDL1 (*r* = −0.36, *P* = 4.23e−8) and (J) PDL2 (*r* = −0.70, *P* = 5.05e−39).

Previous work has highlighted a link between glycolysis and immunosuppression in melanoma^29^ as well as the importance of epigenetic regulation of immune checkpoint expression in various tumor types.^34,35,51,52^ To explore whether these features were relevant in LGG, we first examined the expression of 9 immune checkpoint genes between the glycolysis-high cluster (IDH-wt enriched) and the glycolysis-low cluster (IDH-mutant enriched). Six of these 9 genes showed significantly elevated expression in the glycolysis-high cluster (acceptable *P* < .0055). These genes were PDCD1LG2 (PDL2), PDCD1 (PD1), CD274 (PDL1), IDO1, TNFRSF4 (OX40), and CTLA4 (*P* = 3.90e−18, *P* = 1.67e−15, *P* = 8.08e−09, *P* = 1.10e−4, *P* = 3.49e−6, and *P* = 3.89e−05; Figure 4A–F, respectively). Similar results were observed when the same 6 genes were analyzed in the validation set (PDL2: *P* = 2.50e−28, PD1: *P* = 1.25e−14, PDL1: *P* = 4.56e−06, IDO1: *P* = 5.10e−9, OX40: *P* = 1.77e−05, and CTLA4: *P* = 1.37e−07; Supplementary Figure 3A–F, respectively). When expression analysis of these genes was restricted to the IDH-mutant 1p/19q non-codeletion subtype in the glycolytic-high versus glycolytic-low cluster, expression of 4 immune checkpoint genes remained differentially expressed in both the discovery and validation set (PD-L2, PD1, IDO1, and CTLA4; Supplementary Table 1).

The promoter methylation levels for each of these 6 genes were analyzed next. Of these 6 immune checkpoint genes, PD-L1 and PD-L2 appeared to have the strongest inverse correlations between promoter methylation and gene expression (*r* = − 0.40, *P* = 6.05e−11 and *r* = −0.75, *P* = 4.36e−46, respectively; Figure 4G and H). This relationship appeared more pronounced for PD-L2 in which we also observed a clear separation between the glycolysis-high cluster (orange) and glycolysis-low cluster (blue). Similar results were also observed in the validation set (PDL1: *r* = − 0.36, *P* = 4.23e−8 and PDL2: *r* = −0.70, *P* = 5.05e−39; Figure 4I and J, respectively). In short, patients with lower levels of glycolysis (IDH-mutant enriched cluster of patients), also had higher levels of PD-L1/PD-L2 promoter methylation and correspondingly lower levels of PD-L1 and PD-L2 expression. This relationship was more pronounced for PD-L2 and therefore suggests a strong association between glycolysis and PD-L2 expression perhaps via PD-L2 promoter access in IDH-wild-type patients.

When we analyzed the remaining 4 immune checkpoint genes (IDO1, PD1 [PDCD1], OX40 [TNFRSF4], and CTLA4) that were also elevated in the glycolytic IDH wild-type enriched cluster of LGG samples, weaker/absent associations were observed in both the discovery set (IDO1: *r* = −0.28, *P* = 6.31e−6; PD1: *r* = −0.31, *P* = 5.00e−7; OX40: *r* = −0.27, *P* = 1.45e−5; CTLA4: *r* = −0.12, *P* = .06; Supplementary Figure 4A–D, respectively) and the validation set (IDO1: *r* = −0.34, *P* = 2.69e−8; PD1: *r* = −0.30, *P* = 1.35e−6; OX40: *r* = −0.36, *P* = 4.80e−9; CTLA4: *r* = −0.02, *P* = .72; Supplementary Figure 5 A–D, respectively). These results suggest that the methylation-regulated expression of the immune checkpoint genes analyzed in glycolytic-high (IDH-wild-type-enriched cluster) patients may be relatively specific to PDL1/2.

To determine whether these epigenetic results may be a direct consequence of an inducible IDH mutation, methylation and H3K4me3 enrichment results from 2 IDH-mutant knock-in cell lines (HCT116^50^ and immortalized primary human astrocytes (IHA),^12^ respectively) were examined (Supplementary Table 2, Supplementary Figure 6). Upon examining the enrichment in the overall GO biological processes, sites with elevated methylation in these cell lines as a result of the IDH mutation were found to be enriched for several immune regulation related processes (Supplementary Table 2). In addition, an observed albeit modest decrease in promoter H3K3me4 (marker of active transcription) was observed in LDHA, PDL2, and PDL1 upon induced expression of the IDH mutant, which is in line with our results from human LGG tumor samples (Supplementary Figure 6A–C). Taken together, these findings suggest an epigenetic association between glycolytic expression, IDH-wild-type status, and PDL1/2 expression.

## Discussion

This study features a glycolysis-centered approach to explore the link between metabolic expression, IDH mutation status, and immunosuppressive expression in human LGG samples. We initiated our analysis using an unbiased vector quantization method, and this first revealed a strong association between glycolytic gene expression and wild-type IDH status. These results are in line with the unfavorable clinical features of glycolytic tumors across several tissue types,^53^ and the substantial evidence that pair wild-type IDH LGG patients with poor survival.^2^ Furthermore, while previous work has revealed an association between glycolysis and PD-L1 expression in several cancer types,^54^ PD-L2 has not been explored in the context of glycolysis, nor in the context of human LGG. Interestingly, upon revealing LDHA and PDL2 as 2 specific sites of methylation-associated repression in human LGG, we propose that these genomic observations may be mediated by the neomorphic activity of mutant IDH given its widespread influence on the human DNA methylome.^11,12^

The IDH mutation in LGG has long been known as the hallmark bridge between the genetics and epigenetics of glioma.^11^ While this study allowed us to dive slightly deeper into the pathways associated with these epigenetic changes, it is important to note that glycolysis and immunosuppressive signaling likely mark merely two of the dozens of pathways affected by the IDH-mutant induced methylation.^50^ There are also several limitations to our study. First, the clinical tumor samples analyzed were limited to those of TCGA. This is due to the small amount of public LGG datasets that span multiple the multiple modalities necessary for our study (eg, RNA-seq, DNA methylation, WES, clinical/survival). To this end, it may be interesting to explore whether glycolytic expression associates with patient OS after adjusting for IDH status in a larger multimodal dataset. Second, while our results revealed consistently robust statistical associations, they do not imply a direct cause and effect relationship between IDH mutation, glycolysis, and immune checkpoint expression. Nevertheless, as shown in the previously generated in vitro data from 2 independent groups, the direct epigenetic effect of IDH mutation revealed results in line with our clinical results.^12,50^ Furthermore, although the purpose of our study was to identify clinically associated genomic biomarkers in human LGG, our results extend these previous in vitro findings to show relevance in human tumor data. This may therefore encourage mechanistic exploration of IDH-mutant induced changes in glycolysis and PDL1/2 expression. To this end, there are currently about 40 ongoing clinical trials combining metabolic interventions with immune-checkpoint inhibitors across a wide range of tumors types, suggesting a hopeful shift toward the clinical potential of targeting immunometabolism across various types of human cancers, including glioma.^55^

It has mechanistically been shown that the accumulated 2HG in IDH-mutant gliomas is taken up by T cells and perturbs the activity of nuclear factor of activated T cells and polyamine biosynthesis. This results in the suppression of T cell activity and was shown to be reversible by IDH-mutant inhibition in the context of checkpoint blockade.^56^ Such observations suggest that suppressing the accumulation of 2HG—a metabolite theoretically relatively absent in IDH-wild-type tumors—may directly render LGG cells vulnerable to checkpoint inhibition. The implication of our findings is in line with these observations as PDL1/2+ patients are those who are more likely to respond to checkpoint inhibition and because the IDH-wt tumors that express PDL1/2 are unlikely to contain 2HG. Thus, we believe a potential role for dual checkpoint and glycolytic inhibition in wild-type-IDH LGG may be worthy of further exploration.

While studies examining the association between glycolysis and immunogenic resistance in human tumors are relatively scarce, recent work has shown that tumor glycolytic expression characterizes immune resistance to adoptive T cell therapy in melanoma.^29^ Specifically, increased glycolytic activity was found to impair both T cell-mediated apoptosis and adoptive T cell therapy efficacy, and interestingly, inhibition of LDHA enhanced the antitumor activity of T cells both in vitro and in vivo.^29^ However, it is currently unknown whether this strategy of LDHA inhibition is also beneficial in glioma. In regard to exploring an additional potentially targetable glycolytic driver surfaced directly from our results, it is worth noting the strong association between wild-type IDH patients and hexokinase 2 (HK2) expression. In GBM, HK2 has been shown to be a key mediator of aerobic glycolysis and tumor growth,^57^ which is in line with our observed expression ratio of LDHA/LDHB in the IDH wild-type enriched patient cluster. GBM is thought to uniquely express HK2 as opposed to HK1 in LGG and normal brain tissue. This may suggest that the glycolysis-high cluster (IDH-wt enriched) patient LGGs metabolically behave more similarly to GBM in part via HK2 upregulation. This is further supported by the similar trends in OS between IDH wild-type LGG and GBM.^2^ In terms of the relevant therapeutic implication, previous work has also shown that depletion of HK2 but not HK1 restores oxidative phosphorylation and sensitizes GBM cells to standard therapy.^57^ Thus, in addition to LDHA, HK2 may serve as a potential candidate target for follow-up experimental studies that explore dual inhibition of the immunometabolic interface in glioma.

Our findings are the first to demonstrate not only that a strong association exists between glycolysis, wild-type IDH, and PDL2 expression, but also suggests that glycolytic and PDL2 expression hinge on an epigenetic association that is elevated in IDH-wild-type patients. Moreover, given the documented clinical potential of PD-L2 targeting with pembrolizumab,^27^ further exploration of the mechanistic basis of PD-L2 inhibition in preclinical models of LGG may be warranted. Ultimately, by bridging the genomic correlates of glycolysis, immunosuppression, and IDH mutation status, this work uses several genomic footprints to surface a previously unrecognized immunometabolic association in human LGG.

## Supplementary Material

vdaa162_suppl_Supplementary_Materials
