# Supplementary material for: Glycolytic expression in lower-grade glioma reveals an epigenetic association between IDH mutation status and PDL1/2 expression
Source: Neurooncol Adv. 2020 Nov 27;3(1):vdaa162. doi: 10.1093/noajnl/vdaa162 (PMC7837356; doi:10.1093/noajnl/vdaa162)
Supplement: vdaa162_suppl_Supplementary_Materials [file vdaa162_suppl_supplementary_materials.pptx]

## Slide 1
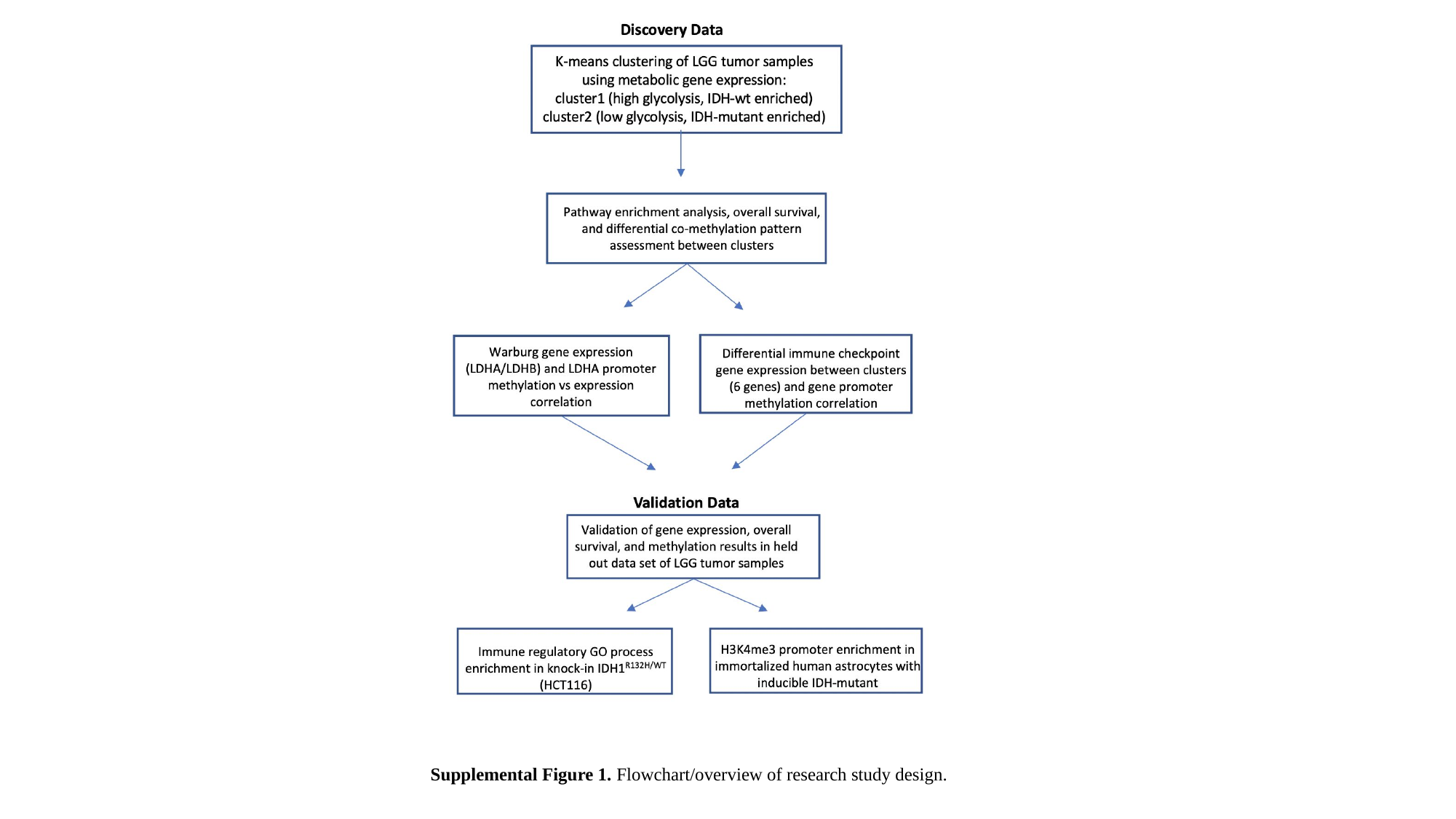

Supplemental Figure 1. Flowchart/overview of research study design.

## Slide 2
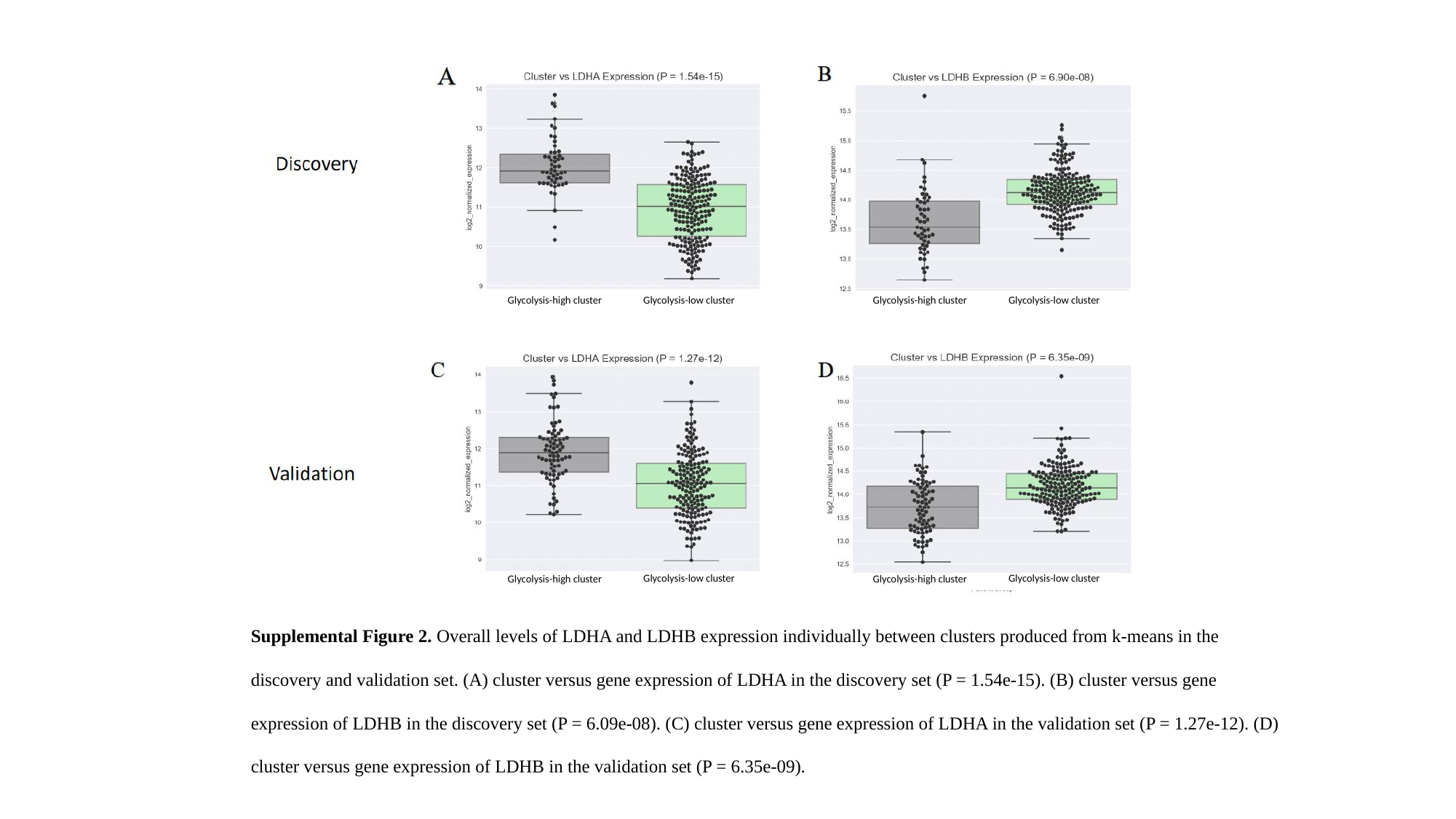

Glycolysis-low cluster
Glycolysis-low cluster
Glycolysis-high cluster
Glycolysis-high cluster
Glycolysis-low cluster
Glycolysis-low cluster
Glycolysis-high cluster
Glycolysis-high cluster
Supplemental Figure 2. Overall levels of LDHA and LDHB expression individually between clusters produced from k-means in the discovery and validation set. (A) cluster versus gene expression of LDHA in the discovery set (P = 1.54e-15). (B) cluster versus gene expression of LDHB in the discovery set (P = 6.09e-08). (C) cluster versus gene expression of LDHA in the validation set (P = 1.27e-12). (D) cluster versus gene expression of LDHB in the validation set (P = 6.35e-09).

## Slide 3
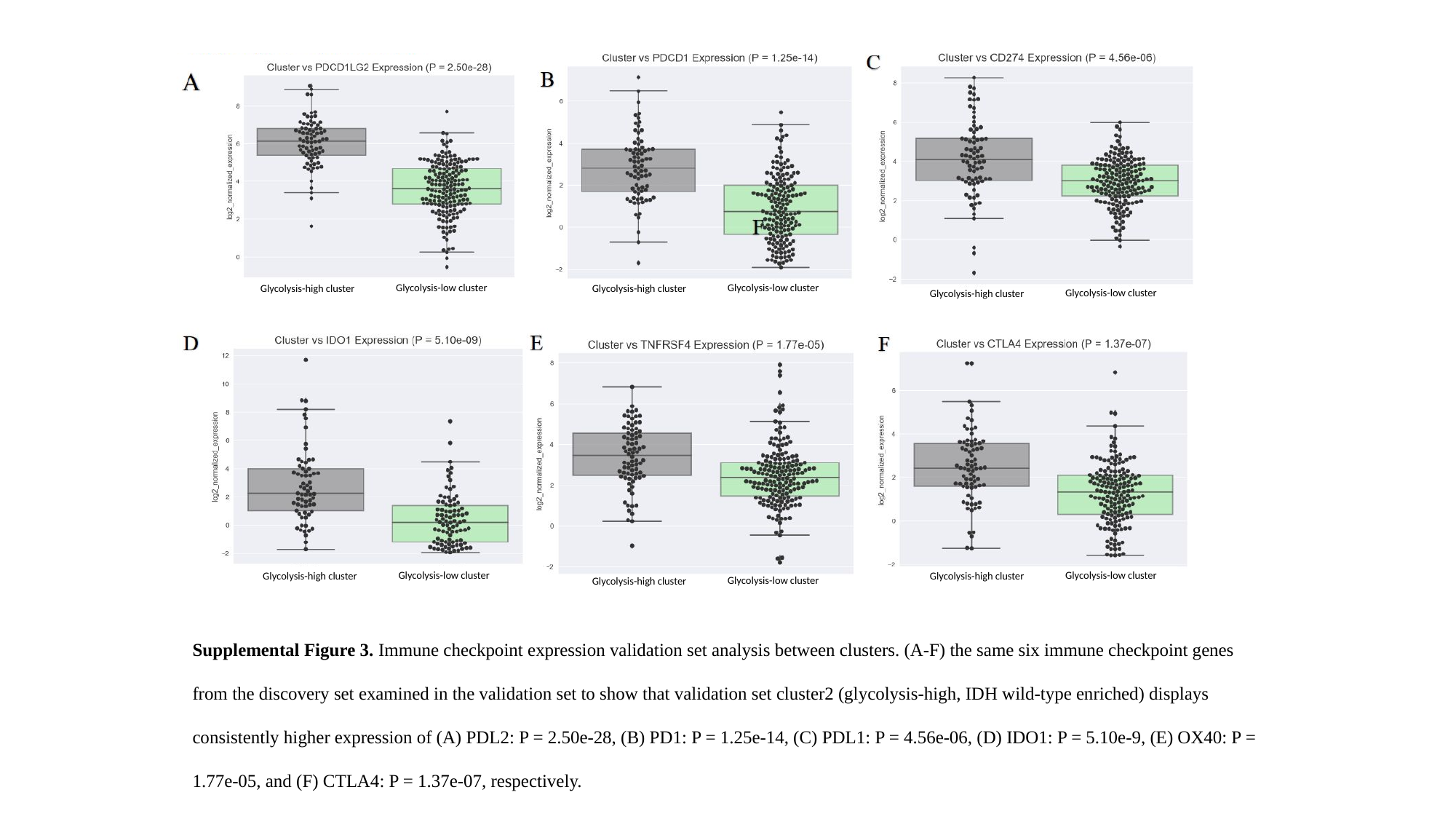

Glycolysis-low cluster
Glycolysis-low cluster
Glycolysis-high cluster
Glycolysis-high cluster
Glycolysis-low cluster
Glycolysis-high cluster
Glycolysis-low cluster
Glycolysis-low cluster
Glycolysis-high cluster
Glycolysis-high cluster
Glycolysis-low cluster
Glycolysis-high cluster
Supplemental Figure 3. Immune checkpoint expression validation set analysis between clusters. (A-F) the same six immune checkpoint genes from the discovery set examined in the validation set to show that validation set cluster2 (glycolysis-high, IDH wild-type enriched) displays consistently higher expression of (A) PDL2: P = 2.50e-28, (B) PD1: P = 1.25e-14, (C) PDL1: P = 4.56e-06, (D) IDO1: P = 5.10e-9, (E) OX40: P = 1.77e-05, and (F) CTLA4: P = 1.37e-07, respectively.

## Slide 4
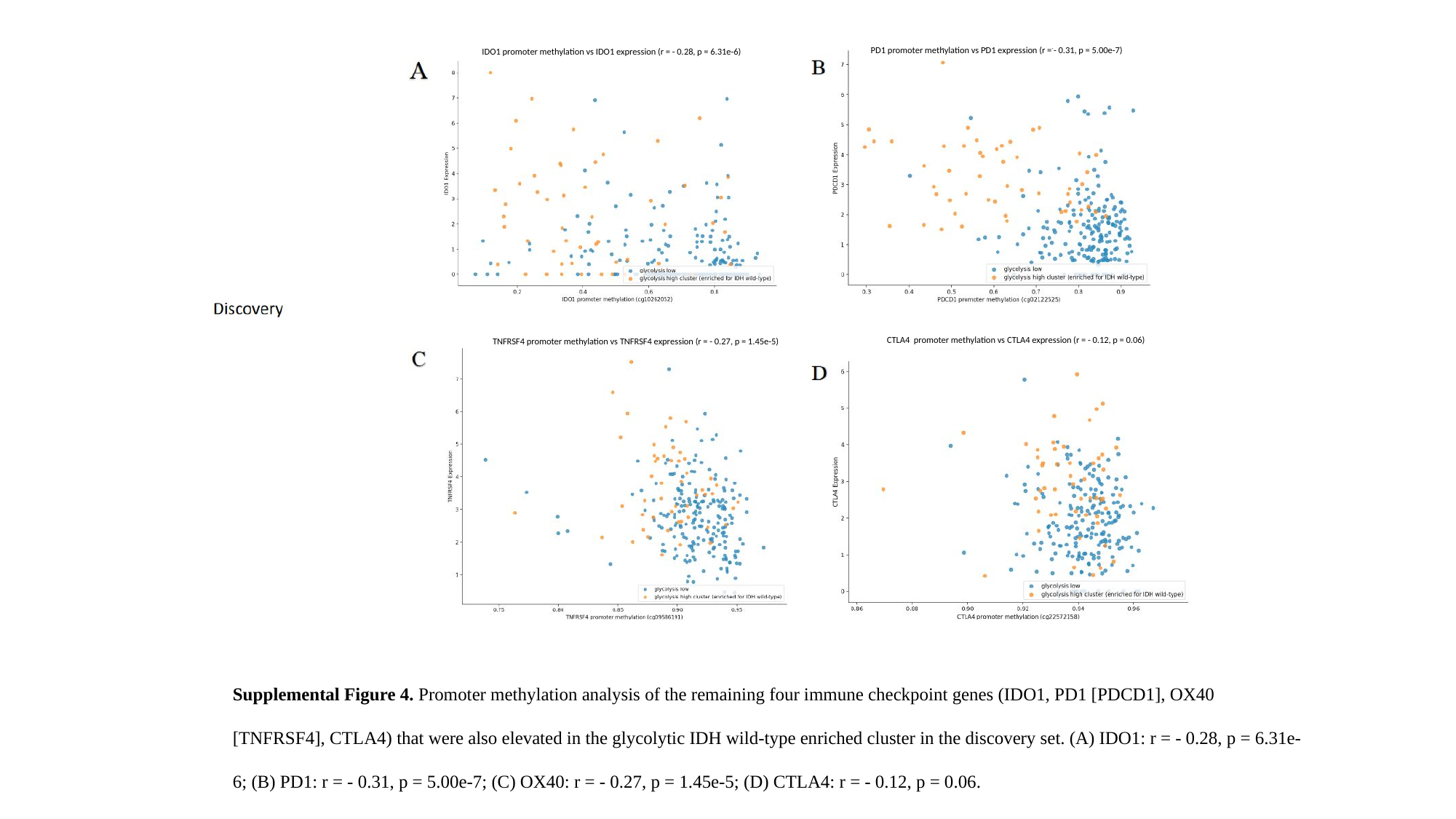

PD1 promoter methylation vs PD1 expression (r = - 0.31, p = 5.00e-7)
IDO1 promoter methylation vs IDO1 expression (r = - 0.28, p = 6.31e-6)
CTLA4 promoter methylation vs CTLA4 expression (r = - 0.12, p = 0.06)
TNFRSF4 promoter methylation vs TNFRSF4 expression (r = - 0.27, p = 1.45e-5)
Supplemental Figure 4. Promoter methylation analysis of the remaining four immune checkpoint genes (IDO1, PD1 [PDCD1], OX40 [TNFRSF4], CTLA4) that were also elevated in the glycolytic IDH wild-type enriched cluster in the discovery set. (A) IDO1: r = - 0.28, p = 6.31e-6; (B) PD1: r = - 0.31, p = 5.00e-7; (C) OX40: r = - 0.27, p = 1.45e-5; (D) CTLA4: r = - 0.12, p = 0.06.

## Slide 5
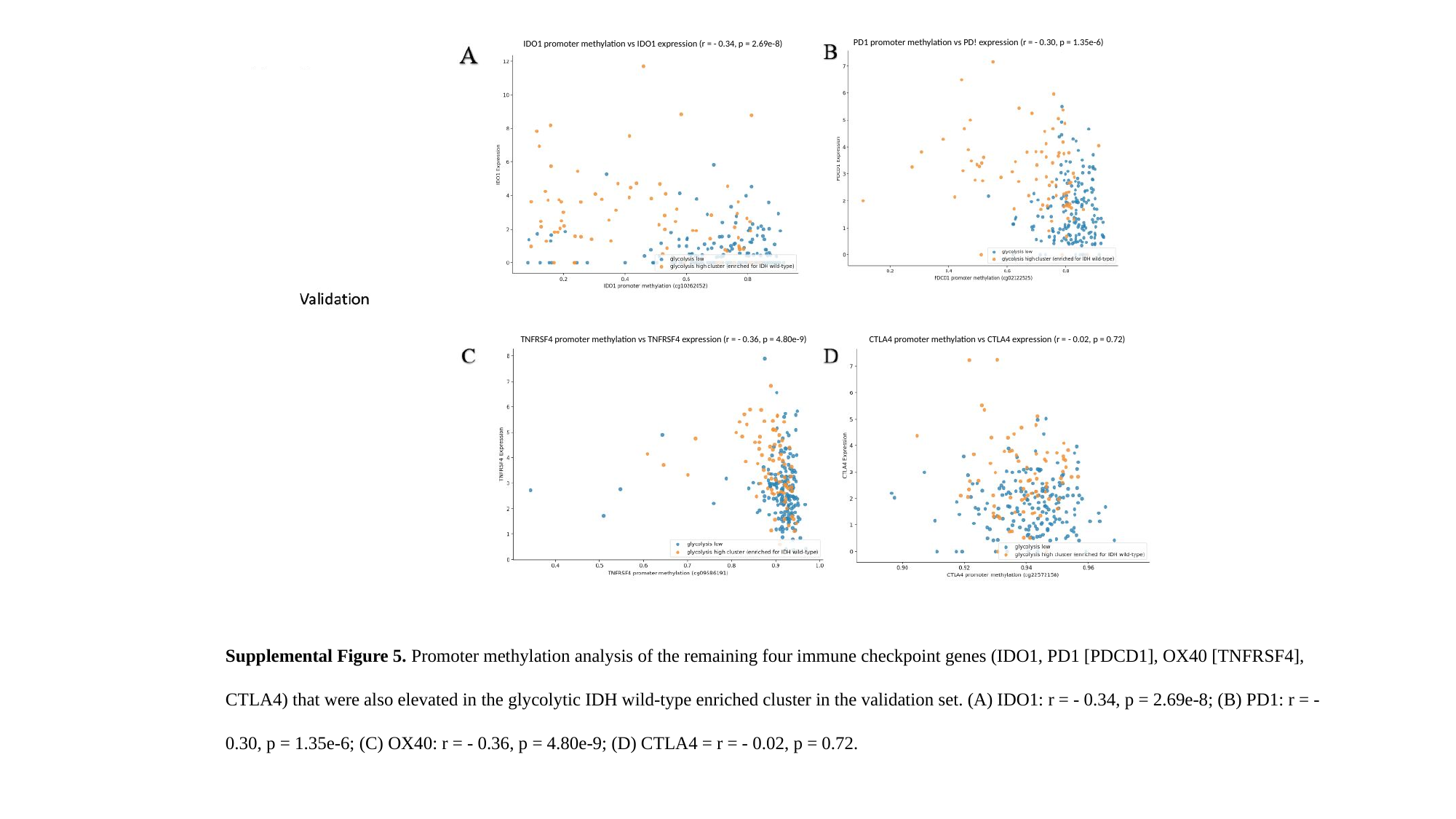

PD1 promoter methylation vs PD! expression (r = - 0.30, p = 1.35e-6)
IDO1 promoter methylation vs IDO1 expression (r = - 0.34, p = 2.69e-8)
TNFRSF4 promoter methylation vs TNFRSF4 expression (r = - 0.36, p = 4.80e-9)
CTLA4 promoter methylation vs CTLA4 expression (r = - 0.02, p = 0.72)
Supplemental Figure 5. Promoter methylation analysis of the remaining four immune checkpoint genes (IDO1, PD1 [PDCD1], OX40 [TNFRSF4], CTLA4) that were also elevated in the glycolytic IDH wild-type enriched cluster in the validation set. (A) IDO1: r = - 0.34, p = 2.69e-8; (B) PD1: r = - 0.30, p = 1.35e-6; (C) OX40: r = - 0.36, p = 4.80e-9; (D) CTLA4 = r = - 0.02, p = 0.72.

## Slide 6
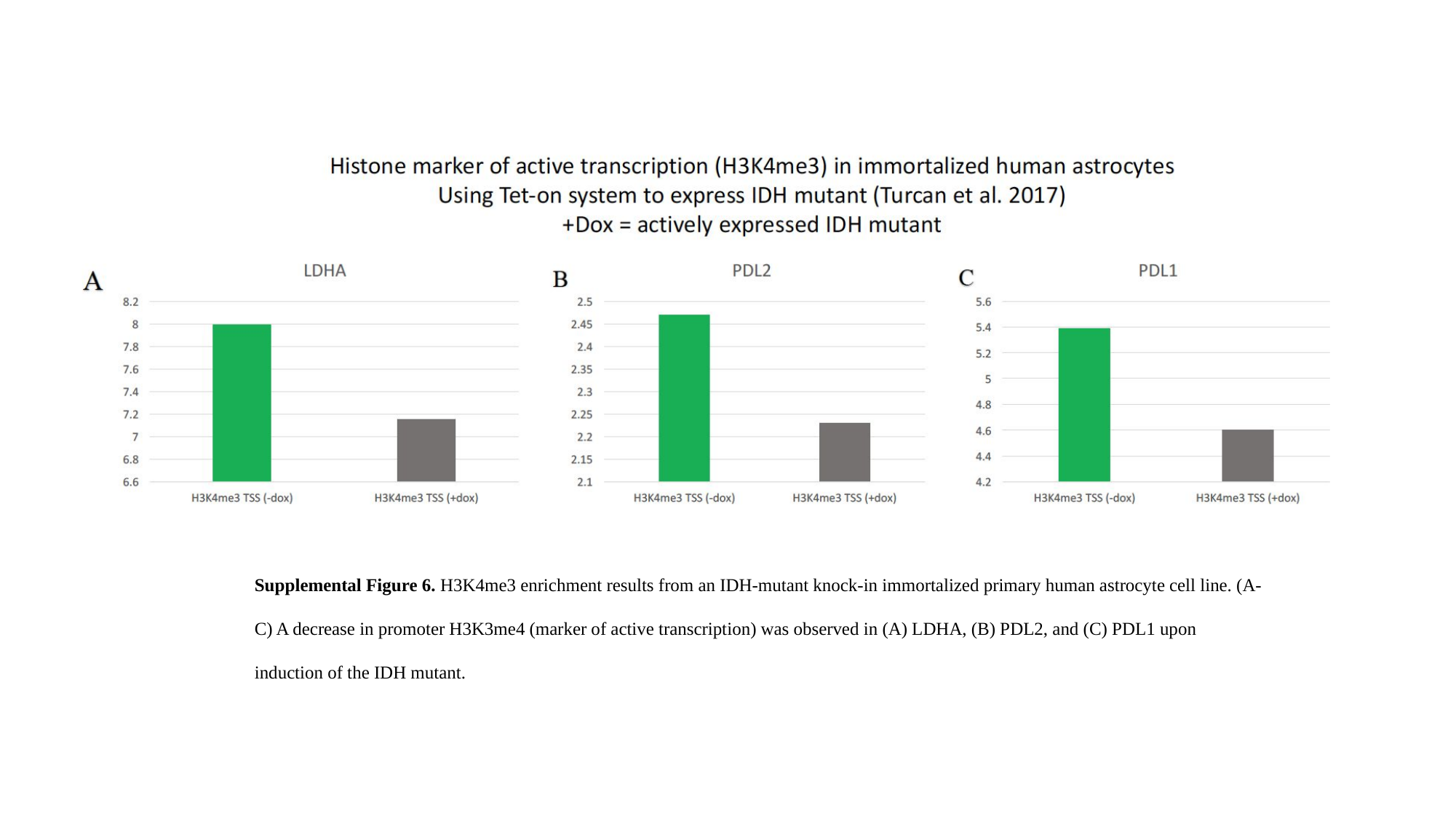

Supplemental Figure 6. H3K4me3 enrichment results from an IDH-mutant knock-in immortalized primary human astrocyte cell line. (A-C) A decrease in promoter H3K3me4 (marker of active transcription) was observed in (A) LDHA, (B) PDL2, and (C) PDL1 upon induction of the IDH mutant.

## Slide 7
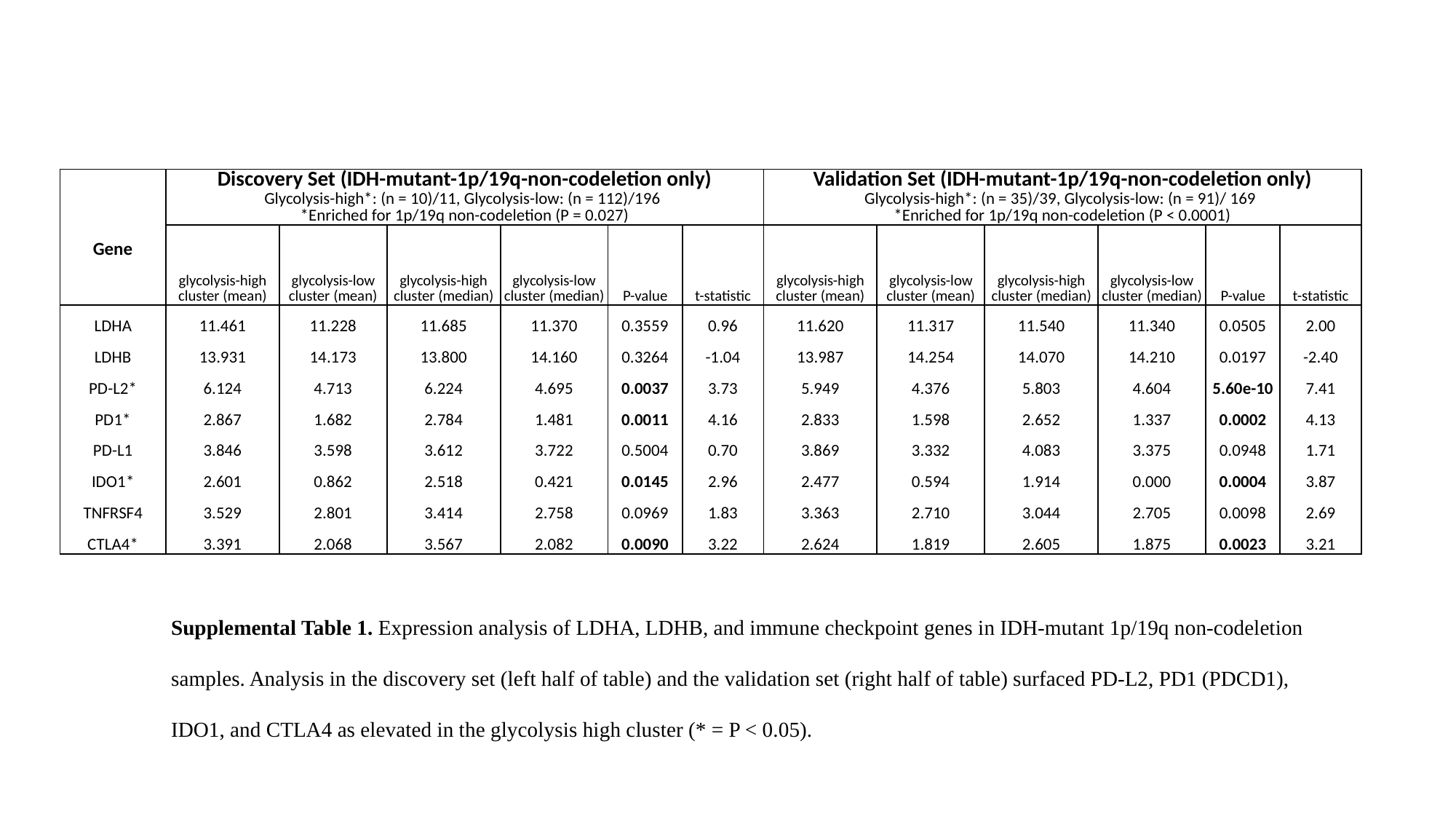

| Gene | Discovery Set (IDH-mutant-1p/19q-non-codeletion only) Glycolysis-high\*: (n = 10)/11, Glycolysis-low: (n = 112)/196 \*Enriched for 1p/19q non-codeletion (P = 0.027) | | | | | | Validation Set (IDH-mutant-1p/19q-non-codeletion only) Glycolysis-high\*: (n = 35)/39, Glycolysis-low: (n = 91)/ 169 \*Enriched for 1p/19q non-codeletion (P < 0.0001) | | | | | |
| --- | --- | --- | --- | --- | --- | --- | --- | --- | --- | --- | --- | --- |
| | glycolysis-high cluster (mean) | glycolysis-low cluster (mean) | glycolysis-high cluster (median) | glycolysis-low cluster (median) | P-value | t-statistic | glycolysis-high cluster (mean) | glycolysis-low cluster (mean) | glycolysis-high cluster (median) | glycolysis-low cluster (median) | P-value | t-statistic |
| LDHA | 11.461 | 11.228 | 11.685 | 11.370 | 0.3559 | 0.96 | 11.620 | 11.317 | 11.540 | 11.340 | 0.0505 | 2.00 |
| LDHB | 13.931 | 14.173 | 13.800 | 14.160 | 0.3264 | -1.04 | 13.987 | 14.254 | 14.070 | 14.210 | 0.0197 | -2.40 |
| PD-L2\* | 6.124 | 4.713 | 6.224 | 4.695 | 0.0037 | 3.73 | 5.949 | 4.376 | 5.803 | 4.604 | 5.60e-10 | 7.41 |
| PD1\* | 2.867 | 1.682 | 2.784 | 1.481 | 0.0011 | 4.16 | 2.833 | 1.598 | 2.652 | 1.337 | 0.0002 | 4.13 |
| PD-L1 | 3.846 | 3.598 | 3.612 | 3.722 | 0.5004 | 0.70 | 3.869 | 3.332 | 4.083 | 3.375 | 0.0948 | 1.71 |
| IDO1\* | 2.601 | 0.862 | 2.518 | 0.421 | 0.0145 | 2.96 | 2.477 | 0.594 | 1.914 | 0.000 | 0.0004 | 3.87 |
| TNFRSF4 | 3.529 | 2.801 | 3.414 | 2.758 | 0.0969 | 1.83 | 3.363 | 2.710 | 3.044 | 2.705 | 0.0098 | 2.69 |
| CTLA4\* | 3.391 | 2.068 | 3.567 | 2.082 | 0.0090 | 3.22 | 2.624 | 1.819 | 2.605 | 1.875 | 0.0023 | 3.21 |
Supplemental Table 1. Expression analysis of LDHA, LDHB, and immune checkpoint genes in IDH-mutant 1p/19q non-codeletion samples. Analysis in the discovery set (left half of table) and the validation set (right half of table) surfaced PD-L2, PD1 (PDCD1), IDO1, and CTLA4 as elevated in the glycolysis high cluster (* = P < 0.05).

## Slide 8
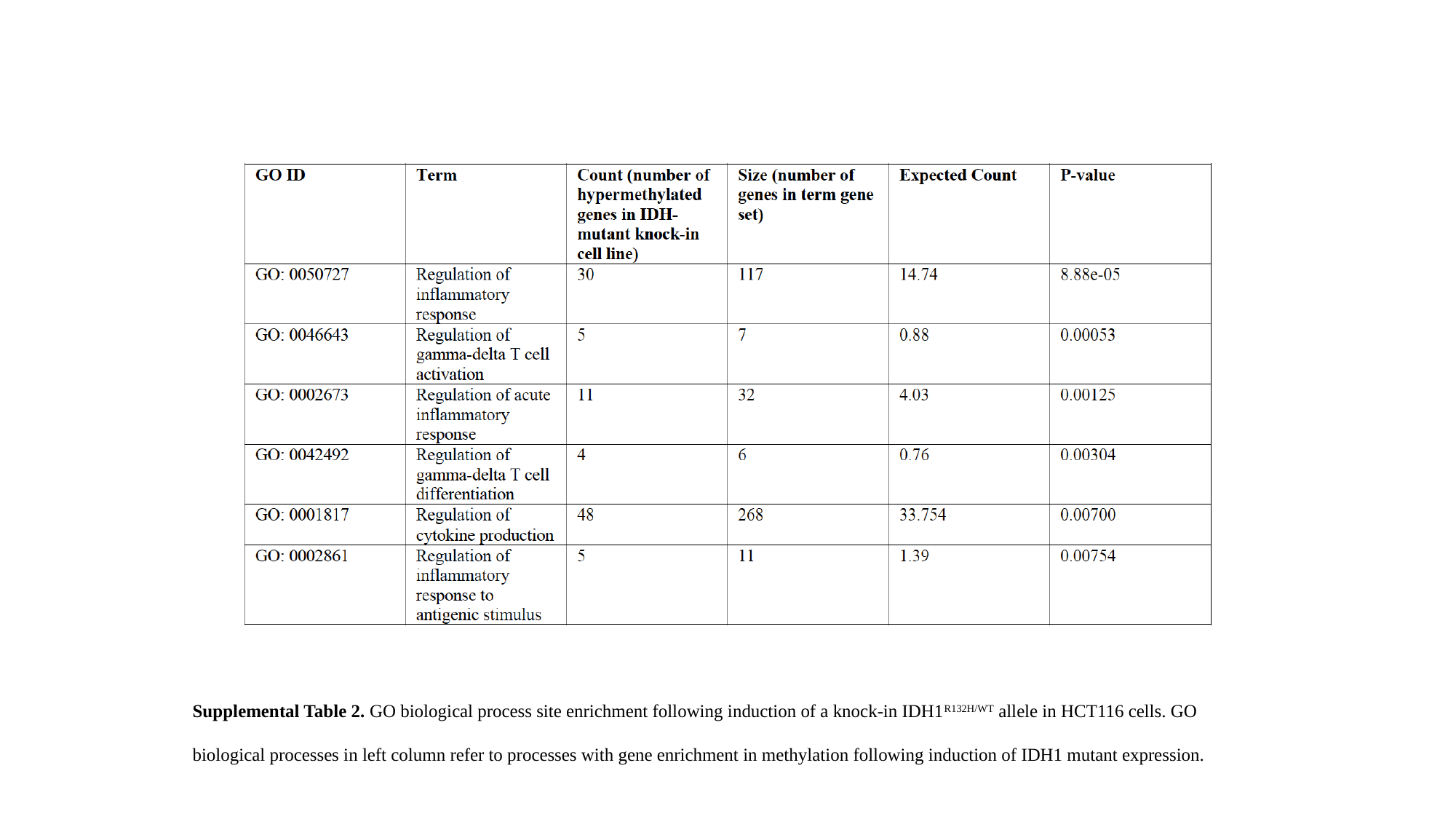

Supplemental Table 2. GO biological process site enrichment following induction of a knock-in IDH1R132H/WT allele in HCT116 cells. GO biological processes in left column refer to processes with gene enrichment in methylation following induction of IDH1 mutant expression.
